# Spatial risk profiling of *Plasmodium falciparum *parasitaemia in a high endemicity area in Côte d'Ivoire

**DOI:** 10.1186/1475-2875-8-252

**Published:** 2009-11-11

**Authors:** Giovanna Raso, Kigbafori D Silué, Penelope Vounatsou, Burton H Singer, Ahoua Yapi, Marcel Tanner, Jürg Utzinger, Eliézer K N'Goran

**Affiliations:** 1 Département Environnement et Santé, Centre Suisse de Recherches Scientifiques, Abidjan, Côte d'Ivoire; 2 School of Population Health, University of Queensland, Brisbane, Australia; 3 Department of Public Health and Epidemiology, Swiss Tropical Institute, Basel, Switzerland; 4 UFR Biosciences, Université de Cocody-Abidjan, Abidjan, Côte d'Ivoire; 5 Office of Population Research, Princeton University, Princeton, USA

## Abstract

**Background:**

The objective of this study was to identify demographic, environmental and socioeconomic risk factors and spatial patterns of *Plasmodium falciparum *parasitaemia in a high endemicity area of Africa, and to specify how this information can facilitate improved malaria control at the district level.

**Methods:**

A questionnaire was administered to about 4,000 schoolchildren in 55 schools in western Côte d'Ivoire to determine children's socioeconomic status and their habit of sleeping under bed nets. Environmental data were obtained from satellite images, digitized ground maps and a second questionnaire addressed to school directors. Finger prick blood samples were collected and *P. falciparum *parasitaemia determined under a microscope using standardized, quality-controlled methods. Bayesian variogram models were utilized for spatial risk modelling and mapping of *P. falciparum *parasitaemia at non-sampled locations, assuming stationary and non-stationary underlying spatial dependence.

**Results:**

Two-thirds of the schoolchildren were infected with *P. falciparum *and the mean parasitaemia among infected children was 959 parasites/*μ*l of blood. Age, socioeconomic status, not sleeping under a bed net, coverage rate with bed nets and environmental factors (e.g., normalized difference vegetation index, rainfall, land surface temperature and living in close proximity to standing water) were significantly associated with the risk of *P. falciparum *parasitaemia. After accounting for spatial correlation, age, bed net coverage, rainfall during the main malaria transmission season and distance to rivers remained significant covariates.

**Conclusion:**

It is argued that a massive increase in bed net coverage, particularly in villages in close proximity to rivers, in concert with other control measures, is necessary to bring malaria endemicity down to intermediate or low levels.

## Background

Malaria remains one of the most pressing public health and poverty-related issue in the developing world, particularly in sub-Saharan Africa [1]. Each year, malaria might claim the lives of >1 million individuals. There are >500 million episodes of clinical *Plasmodium falciparum *malaria and the global burden might exceed 40 million disability-adjusted life years (DALYs) [2-4]. Mortality, morbidity and economic losses due to malaria could be reduced significantly if effective measures, such as sleeping under long-lasting insecticidal nets (LLINs) and access to prompt diagnosis and effective treatment using artemisinin-based combination therapy (ACT) were made available to all those in need [5]. Interventions aiming at the control and local elimination of malaria require reliable risk maps in order to enhance the efficacy and cost-effectiveness of control measures. Since parasitaemia is correlated with clinical manifestations of malaria [6], parasitaemia risk maps are a useful tool for the spatial targeting of control interventions. Ongoing blood sampling at the household level on a broad scale is expensive and not practical for surveillance purposes. District-level planning and targeting would be greatly facilitated by rapid and non-invasive identification of high-risk zones.

Over the past decade, geographical information system (GIS) and remote sensing technologies have been widely used for mapping malaria [1,7,8]. However, purely GIS and remote sensing approaches have a number of shortcomings, due to their inability to quantify the relation between environmental factors and malaria risk and, consequently, infer predictions from statistical models [9]. Furthermore, classical statistical models have been widely employed to evaluate the relationship between disease risk and demographic, environmental and socioeconomic factors, assuming independence of spatially-explicit data [10-12]. Since disease data cluster in space, the assumption of independence is violated, and hence the statistical significance of the model covariates often overestimated [13]. It follows that predictive risk models lack accuracy.

In recent work by the authors, Bayesian non-stationary geostatistical models were employed for spatial risk profiling of malaria [9,14,15]. The strengths of these models are their accountancy for spatial dependence in the data, and the assumption of non-stationary spatial processes. The use of non-stationary models is further justified on the ground that local characteristics related to human behaviour and environment, including vector ecology, depend on location. Consequently, assuming stationarity may provide unreliable results when analyzing spatially-explicit disease data.

Here, risk factors and spatial patterns of *P. falciparum *parasitaemia among school-aged children in a high endemicity setting of western Côte d'Ivoire are elucidated. An integrated approach, using GIS and remotely-sensed environmental data, questionnaire and parasitological survey data and Bayesian geostatistical models was employed. Finally, the use of non-stationary models for risk profiling of *P. falciparum *parasitaemia at a regional scale was explored. The identified risk factors can help district health planners to implement malaria control interventions in a spatially-explicit manner, followed by monitoring and surveillance so that control tools can be fine-tuned over time to enhance their performance [16].

## Methods

### Study area and population

This study was carried out in the region of Man, a mountainous area in the western part of Côte d'Ivoire, which is highly endemic for *P. falciparum *malaria, as well as helminth infections [17-21]. Climate conditions are tropical with rains occurring from March to October with highest precipitation observed in July and August. The dry season extends from November to February.

The present study was carried out between October 2001 and February 2002. Schoolchildren from 57 rural schools attending grades 3-5 were invited for finger prick blood samples and two questionnaires were administered, one addressed to schoolchildren and the second one to school directors.

### Ethical clearance

The study protocol was approved by the institutional research commissions of the Swiss Tropical Institute (Basel, Switzerland) and the Centre Suisse de Recherches Scientifiques (Abidjan, Côte d'Ivoire). Ethical clearance was obtained by the Ministry of Health in Côte d'Ivoire.

### Cross-sectional surveys

Thin and thick blood films were prepared from finger prick blood samples on microscope slides, air-dried and transferred to a laboratory in the town of Man. Slides were stained with 10% Giemsa and examined under a light microscope by experienced laboratory technicians. The number of *Plasmodium *spp. parasites was counted by assuming a standard white blood cell count of 8,000/*μ*l of blood.

The schoolchildren questionnaire was used to obtain information about assets on ownership and household characteristics (total of 12 indicators), and perceived symptoms and diseases (total of 17 morbidity indicators). In addition, children were asked whether they slept under a bed net and whether they were living in the village of the school or in a nearby village or hamlet. An asset-based approach was used to stratify schoolchildren into five socio-economic groups [19].

The questionnaire addressed to school directors included three main topics, i.e., (i) village demographics, (ii) health issues and (iii) local environment (e.g., presence of swamps, irrigation fields and pasture nearby the village and the estimated distances). In case the school directors felt they were not sufficiently acquainted to respond to these questions, they were invited to consult with other village authorities.

### Environmental data

Geographical coordinates for each school were collected using a hand-held Magellan 320 global positioning system (GPS; Thales Navigation, Santa Clara, CA, USA). Distance to rivers was calculated from digitized ground maps. Normalized difference vegetation index (NDVI) and land surface temperature (LST) were downloaded at 1 × 1 km spatial resolution from Moderate Resolution Imaging Spectroradiometer (MODIS) from USGS EROS Data Centre. Rainfall estimate (RFE) data with an 8 × 8 km spatial resolution from Meteosat 7 satellite were obtained from the Africa Data Dissemination Service (ADDS). NDVI, LST and RFE were downloaded for the period of September 2001 to August 2002 and processed as detailed elsewhere [22]. A digital elevation model (DEM) was employed originating from the Shuttle Radar Topography Mission (SRTM) to delineate watersheds [23].

### Data management and analysis

Data were entered twice and cross-checked. Geographical data were displayed in ArcView GIS version 3.2 (Environmental Systems Research Institute, Inc., Redlands, CA, USA). Schoolchildren were subdivided into two age groups: (i) 6-10 years and (ii) 11-16 years. Bed net coverage was calculated as the percentage of schoolchildren who reported sleeping under a bed net at the unit of the school.

All demographic, environmental and socioeconomic covariates were fitted into negative binomial regression models on the *P. falciparum *parasitaemia data, using STATA version 9.0 (Stata Corporation, College Station, TX, USA). Covariates with a significance level <0.15 were built into three different spatial models for *P. falciparum *parasitaemia using WinBUGS version 1.4 (Imperial College & Medical Research Council, London, UK). The models were (i) a stationary Bayesian negative binomial regression model, and (ii) two non-stationary Bayesian negative binomial regression models. To take into account the spatial heterogeneity, location-specific random effects were integrated in the logistic models, assuming that they are distributed according to a multivariate normal distribution with variance-covariance matrix related to the variogram of the spatial process. Markov chain Monte Carlo (MCMC) simulation was employed to estimate the model parameters [24]. Model covariates from the final model were utilized to generate a smooth map of *P. falciparum *parasitaemia using Bayesian kriging [25].

### Model specification

To model *P. falciparum *parasitaemia, let *Z*_*ij *_be the *P. falciparum *parasite count in blood films of schoolchild *j *in village *i*. It was assumed that *Z*_*ij *_arises from a negative binomial distribution, *Z*_*ij*_~*Nb*(*μ*_*ij*_, *r*) with mean *μ*_*ij *_and over-dispersion (extra variation) *r*. The covariates *X*_*ij *_and village-specific random effect *ϕ*_*i *_were modeled with log(*μ*_*ij*_) as the outcome, that is log(*μ*_*ij*_) = *β *+ *ϕ*_*i*_, where *β *is the vector of regression coefficients. The spatial correlation was introduced on the *ϕ*_*i*_'s by assuming that *ϕ * = (*ϕ*_1_, *ϕ*_2_, ..., *ϕ*_*N*_)^*T *^has a multivariate normal distribution, *β * ~*MVN*(0, Σ), with variance-covariance matrix Σ. Moreover, an isotropic spatial process was assumed, i.e., Σ_*mn *_= *σ*^2 ^exp(-*ud*_*mn*_), where *d*_*mn *_is the Euclidean distance between village *m *and village *n*, *σ*^2 ^is the geographic variability known as the sill, and *u *is a smoothing parameter that controls the rate of correlation decay with increasing distance. To take into account non-stationarity, the study area was partitioned in *K *subregions and a local stationary spatial process  was assumed in each subregion *k = *1, ..., *K*. One type of model included ecological subregions, i.e., watersheds of rivers, whereas the other type included fixed subregions, i.e., the study area was subdivided into two subregions on a diagonal from the north-western corner to the south-eastern corner of the study area. Spatial correlation in the study area was viewed as a mixture of the different spatial processes. The spatial random effect *ϕ*_*i *_at location *i *was modeled as a weighted average of the subregion-specific (independent) stationary processes as follows: , with weights *a*_*ik*_, which are decreasing functions of the distance between location *i *and the centroids of the subregions *k *[26]. Assuming *ω*_*k *_~*MVN*(0, Σ_*k*_), , it follows that , with *A*_*k *_= *diag*{*a*_1*k*_, *a*_2*k*_, ..., *a*_*nk*_}. The range is defined as the minimum distance at which spatial correlation between locations is below 5%. It can be calculated as  and is expressed in meters.

### Model implementation

Following a Bayesian model specification, prior distributions for the model parameters were adopted. Vague Normal distributions for the *β *parameters with large variances (i.e., 10,000), gamma prior for *r *with large variance, inverse gamma priors for  and uniform priors for *u*_*k*_, *k *= 1, ..., *K *were chosen. MCMC simulation was employed to estimate the model parameters [24]. A single chain sampler with a burn-in of 5,000 iterations was run. Convergence was assessed by inspection of ergodic averages of selected model parameters. Covariates from the binomial regression models were used to generate a smooth risk map for *P. falciparum *parasitaemia using Bayesian kriging [25].

### Model performance and predictive ability

The deviance information criterion (DIC) was utilized to assess the model performance [27]. Additionally, a two-stage approach was adapted for assessment of model performance based on the predictive ability. First, a training sample from the current database was utilized by fitting individual-level data from 43 randomly selected schools into the negative binomial regression models. The individual-level data from the remaining schools were utilized for prediction purposes. 95%, 75%, 50%, 25% and 1-5% Bayesian credible intervals (BCIs) of the posterior predictive distribution of test individuals were calculated. The model with the highest percentage of correctly predicted individual parasitaemia within the interval with the smallest coverage was considered as the best predicting one. Second, the predictive ability of the models was assessed using a Bayesian p-value analogue calculated from the predictive posterior distribution, recently presented by Gosoniu and colleagues [9]. The Bayesian p-value is calculated as . *I*(·) denotes the number of points fulfilling the specific condition in the argument, *p*_*i*_^*obs *^is the observed parasitaemia of an individual and *p*_*i*_^*rep *^= *p*_*i*_^*rep*(1)^,..., *p*_*i*_^*rep*(1000) ^are 1,000 replicated data from the predictive distribution for a test individual. When the median of the predictive posterior distribution is close to 0.5, the model predicts the observed data well. The model with median p-values closest to 0.5 is considered the best performing one.

## Results

### Study cohort

A total of 3,962 schoolchildren had complete data records, i.e., were individually interviewed and had *P. falciparum *parasitaemia results from blood film examination. There were 2,340 boys (59.1%) and 1,622 girls (40.9%). With regard to age, 1,684 children (42.5%) were between 6 and 10 years, whereas 2,278 children (57.5%) were aged 11-16 years.

### Plasmodium falciparum parasitaemia

Almost two out of three children were infected with *P. falciparum *(64.9%). Other *Plasmodium *species were rare: *Plasmodium malariae *and *Plasmodium ovale *infections were found in 117 (3.0%) and 7 children (0.2%), respectively. All subsequent analyses focus on *P. falciparum*. At the unit of the school, the prevalence of *P. falciparum *ranged from 34.0% to 91.9%.

Among *P. falciparum*-infected children, the mean parasitaemia was 959 parasites/*μ*l of blood. Whilst approximately a third of the children had no *P. falciparum *infection as determined by light microscopy, a third of the children had a *P. falciparum *parasitaemia <500 parasites/*μ*l of blood (37.9%), and one-fourth had a parasitaemia ranging between 500 and 5,000 parasites/*μ*l of blood (25.1%). Only 72 (1.8%) of the children had a parasite count >5,000 parasites/*μ*l of blood. At the unit of the school, the mean *P. falciparum *parasitaemia ranged from 63 to 2,178 parasites/*μ*l of blood.

### Risk profiling and spatial patterns

Results of the bivariate non-spatial analyses are shown in Table 1. Children aged 6-10 years were at a significantly higher risk of having a high *P. falciparum *parasitaemia than their older peers. Sex was not significantly associated with *P. falciparum *parasitaemia. Children from the fourth and fifth quintile (the less poor and least poor) were at a higher risk of having higher parasitaemia levels compared to the poorest schoolchildren. Other significant risk factors included not sleeping under a bed net, bed net coverage at the unit of school, NDVI, RFE, LST, close proximity to standing water (rivers, swamps and irrigated fields) and absence of pasture near villages. There was no significant association between *P. falciparum *parasitaemia and distance to the closest health care facility. Finally, no significant association was found between *P. falciparum *parasitaemia and the children's place of residence (living in the same village as the school or in a nearby village or hamlet).

**Table 1:** Results of the bivariate negative binomial regression models for *P. falciparum *parasitaemia among 3,962 schoolchildren from 55 rural schools of western Côte d'Ivoire.

Source of data	Indicator	*Plasmodium falciparum *parasitaemia		
		
		IRR^a^	95% CI^b^	*P*-value (AIC^c^)
School registry	Age			
	6-10 years	1.00		
	11-16 years	0.70	0.60, 0.83	<0.001
Questionnaire addressed to schoolchildren	Socioeconomic status			
	Most poor	1.00		
	Very poor	1.02	0.79, 1.32	
	Poor	0.93	0.72, 1.20	
	Less poor	1.34	1.04, 1.73	
	Least poor	1.34	1.04, 1.73	0.005
	Sleeping under a bed net	0.75	0.58, 0.98	0.040
	Bed net coverage			
	<25%	1.00		
	≥ 25%	0.38	0.28, 0.53	<0.001
	Living in the same village as school	0.86	0.70, 1.06	0.162
Health district registry	Distance to health care facility	1.00	0.93, 1.08	0.916
Questionnaire addressed to school directors	Presence of swamps for rice cultivation	0.85	0.72, 1.01	0.124
	Distance to irrigated field			
	<500 m	1.00		
	500-999 m	0.55	0.32, 0.96	
	≥ 1000 m	0.86	0.59, 1.22	0.117
	Presence of pasture	0.77	0.62, 0.96	0.022
Satellite images	NDVI			
	Mean I^§^	1.03	0.94, 1.12	0.532 (46,255)
	Mean II^¶^	1.06	0.97, 1.15	0.177 (46,253)
	Mean III^||^	0.95	0.88, 1.03	0.205 (46,254)
	Annual mean	1.00	0.92, 1.08	0.950 (46,255)
	Mean of the transmission season	1.14	1.05, 1.23	0.001 (46,245)
	Annual mean NDVI (categorized)			
	<0.65	1.00		
	0.65-0.70	1.66	1.30, 2.12	
	>0.70	1.31	1.05, 1.64	<0.001 (46,240)
	RFE			
	Mean I^d^	1.06	0.98, 1.15	0.122 (46,253)
	Mean II^e^	1.01	0.94, 1.09	0.742 (46,255)
	Mean III^f^	1.11	1.02, 1.20	0.015 (46,249)
	Sum of annual rainfall	1.17	1.08, 1.26	<0.001 (46,238)
	Mean of the transmission season^g^	1.22	1.13, 1.32	<0.001 (46,230)
	Maximum LST	1.09	1.00, 1.18	0.048
Digitized ground maps	Distance to rivers	0.85	0.78, 0.92	<0.001 (46,241)
	Distance to rivers (categorized)			
	<500 m	1.00		
	500-999 m	0.98	0.80, 1.21	
	≥ 1000 m	0.62	0.51, 0.76	< 0.001 (46,236)

^a^IRR: incidence-rate ratio; ^b^CI: confidence interval; ^c^AIC: Akaike information criterion; ^d^Mean I: mean value during the month prior to blood sample collection; ^e^Mean II: mean value during the month of collection and the previous month; ^f^Mean III: mean value during the month of collection and the two previous months; ^g^The transmission season is during June to August

**Figure 1:**
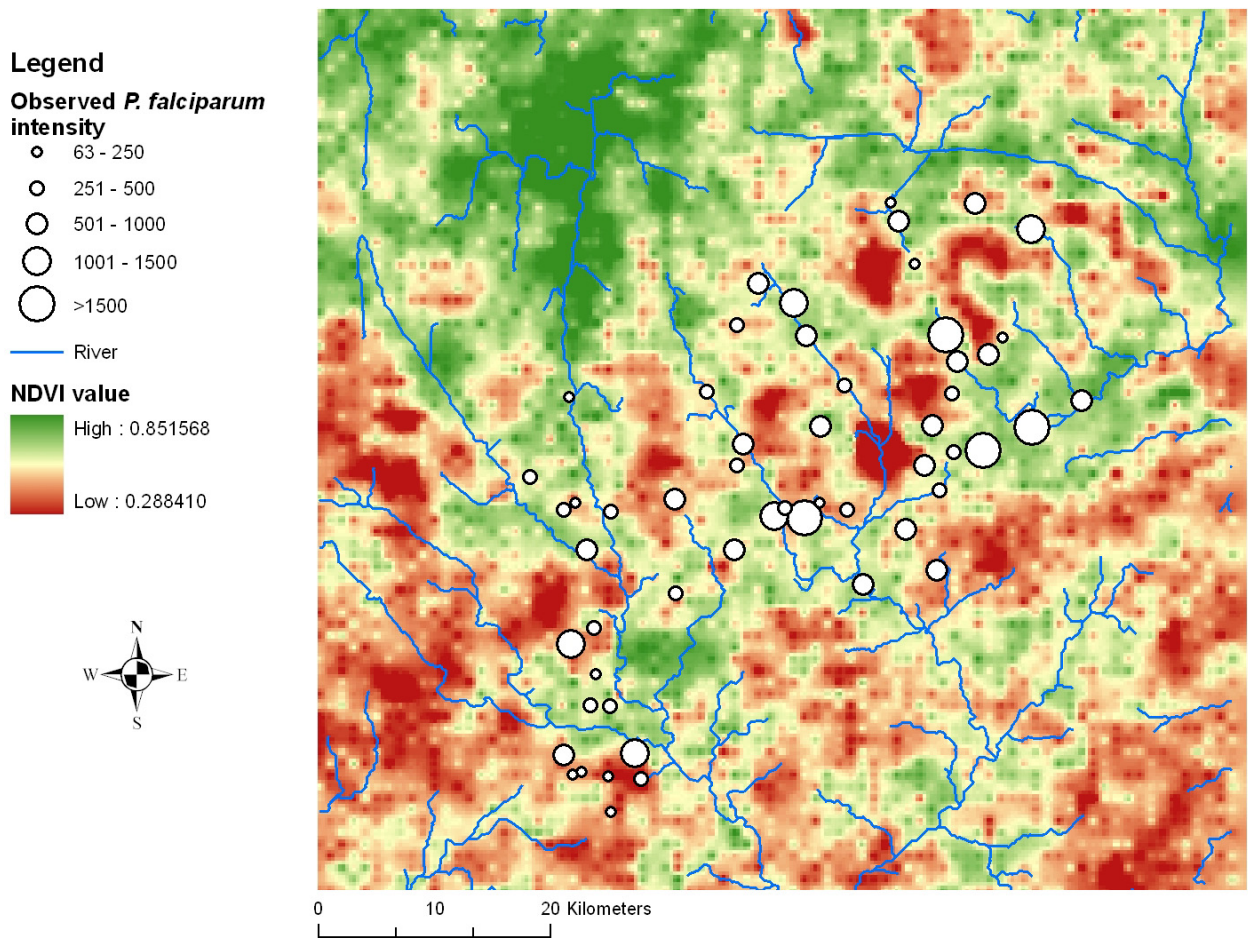
**Mean *P. falciparum *parasitaemia among 3,962 schoolchildren from 55 sampled schools in the region of Man, western Côte d'Ivoire during the school year 2001/2002**. The normalized difference vegetation index (NDVI) is displayed in the background.

The mean *P. falciparum *parasitaemia at the unit of the school is shown in Figure 1. Three schools in the north-eastern part of the study area and one in the central part had a mean parasitaemia >1,500 parasites/*μ*l of blood.

### Spatial analyses and model performance

Results of the spatial analyses are summarized in Table 2. Children's age, bed net coverage and mean RFE during the main malaria transmission season (June to August) were significant covariates in the stationary negative binomial regression model. The three covariates were also found significant in the non-stationary model with ecological subregions. In contrast, mean RFE during the transmission season was not significant in the negative binomial regression model with fixed subregions. In the latter model, age, bed net coverage and distance to rivers were significant covariates. There was a clear over-dispersion (*r *= 0.16) of the data. The range where spatial correlation is below 5% was 1.9 km in the stationary model. For the non-stationary model with ecological subregions, the ranges were 2.3 km, 1.9 km and 2.1 km, respectively. For the non-stationary model with fixed subregions, the ranges were 1.9 km and 2.3 km. Geographical variability differed depending on the subregion in the non-stationary model with ecological subregions.

**Table 2:** Multivariate stationary and non-stationary spatial analyses results for *P. falciparum *parasitaemia for the region of Man, western Côte d'Ivoire.

Indicator	Bayesian negative binomial regression models					
	
	Stationary		Non-stationary with ecological subregions		Non-stationary with fixed subregions	
	
	IRR^a^	95% BCI^b^	IRR^a^	95% BCI^b^	IRR^a^	95% BCI^b^
Age (years)						
6-10	1.00		1.00		1.00	
11-16	0.72	0.60, 0.85	0.71	0.60, 0.83	0.71	0.59, 0.83
Socioeconomic status						
Most poor	1.00		1.00		1.00	
Very poor	1.02	0.77, 1.33	1.00	0.75, 1.29	1.01	0.77, 1.30
Poor	0.97	0.74, 1.26	0.96	0.73, 1.24	0.97	0.74, 1.25
Less poor	1.14	0.85, 1.51	1.09	0.82, 1.43	1.10	0.83, 1.43
Least poor	1.08	0.79, 1.44	1.04	0.76, 1.39	1.03	0.76, 1.37
Sleeping under a bed net	0.86	0.63, 1.14	0.88	0.65, 1.17	0.87	0.65, 1.16
Bed net coverage (%)						
< 25	1.00		1.00		1.00	
≥ 25	0.51	0.24, 0.98	0.49	0.27, 0.82	0.50	0.26, 0.90
Presence of swamps for rice cultivation	1.11	0.71, 1.63	1.23	0.80, 1.77	1.13	0.73, 1.67
Distance to irrigated field (m)						
< 500	1.00		1.00		1.00	
500-999	0.74	0.17, 2.23	0.66	0.23, 1.48	0.64	0.21, 1.59
≥ 1000	0.92	0.35, 2.14	0.95	0.47, 1.74	0.89	0.39, 1.76
Presence of pasture	0.99	0.57, 1.59	0.88	0.57, 1.32	0.92	0.58, 1.43
NDVI (categorized)						
< 0.65	1.00		1.00		1.00	
0.65-0.70	1.42	0.81, 2.43	1.57	0.95, 2.40	1.61	0.97, 2.49
> 0.70	0.76	0.42, 1.33	0.78	0.46, 1.24	0.78	0.46, 1.24
Mean rainfall during transmission season	1.28	1.00, 1.61	1.24	1.01, 1.51	1.20	0.95 1.54
Maximum LST	0.93	0.75, 1.15	0.95	0.76, 1.15	0.93	0.72, 1.16
Distance to rivers (m)						
< 500	1.00		1.00		1.00	
500-999	1.13	0.68, 1.74	1.08	0.73, 1.56	1.03	0.66, 1.56
≥ 1000	0.72	0.44, 1.09	0.71	0.46, 1.08	0.63	0.42, 0.94
*r*^c^	0.16	0.16, 0.17	0.16	0.16, 0.17	0.16	0.16, 0.17
*ρ*_1_^d^	0.0016	0.0005, 0.003	0.0014	0.0002, 0.002	0.0013	0.0002, 0.002
*ρ*_2_			0.0016	0.0005, 0.003	0.0016	0.0005, 0.003
*ρ*_3_			0.0013	0.0002, 0.002		
*σ*_1_^2e^	0.33	0.17, 0.57	0.14	0.02, 0.39	0.13	0.02, 0.39
*σ*_2_^2^			1.20	0.40, 3.08	0.58	0.26, 1.16
*σ*_3_^2^			0.21	0.03, 0.60		
**DIC^f^**	**46,047.4**		**46,044.9**		**46,045.3**	

^a^IRR: incidence-rate ratio; ^b^BCI: Bayesian credible interval; ^c^*r*: over-dispersion parameter; ^d^*ρ*: scalar parameter representing the rate of decline of correlation with distance between points; ^e^*σ*^2^: estimate of geographic variability; ^f^DIC: deviance information criterion; a composite measure of how well the model does, i.e. a compromise between fit and complexity, with smaller DICs indicating better performance of the model.

For the assessment of the model performance, the spatial models without the covariates bed net coverage, presence of swamps, distance to irrigated fields and presence of pasture were used, since no information was available for prediction. Results of the spatial analyses of those models are shown in Table 3. The differences between DICs for the three models were only marginal, and hence the results suggest that the stationary and the non-stationary models performed similarly. Table 4 summarizes the results of the models' predictive ability using different BCIs. Virtually no difference was found between the stationary and the non-stationary models, although the latter type of models seemed to perform slightly better at the smallest BCIs. The p-values calculated from the predictive distribution of the 1,034 selected individuals for model validation revealed similar distributions for all three models, including medians, suggesting that the models had the same predictive ability.

**Table 3:** Multivariate stationary and non-stationary spatial analyses results for *P. falciparum *parasitaemia for the region of Man, western Côte d'Ivoire.

Indicator	Bayesian negative binomial regression models					
	
	Stationary		Non-stationary with ecological sub-regions		Non-stationary with fixed sub-regions	
	
	IRR^a^	95% BCI^b^	IRR^a^	95% BCI^b^	IRR^a^	95% BCI^b^
Age (years)						
6-10	1.00		1.00		1.00	
11-16	0.72	0.60, 0.85	0.71	0.60, 0.83	0.71	0.59, 0.83
Socioeconomic status						
Most poor	1.00		1.00		1.00	
Very poor	1.02	0.77, 1.33	1.00	0.75, 1.30	1.00	0.76, 1.23
Poor	0.98	0.74, 1.27	0.96	0.73, 1.25	0.96	0.73, 1.22
Less poor	1.15	0.87, 1.52	1.11	0.84, 1.46	1.11	0.83, 1.44
Least poor	1.09	0.80, 1.46	1.07	0.79, 1.42	1.05	0.77, 1.39
Sleeping under a bed net	0.81	0.60, 1.07	0.81	0.61, 1.07	0.82	0.61, 1.08
NDVI (categorized)						
<0.65	1.00		1.00		1.00	
0.65-0.70	1.44	0.84, 2.35	1.54	0.94, 2.36	1.69	1.04, 2.54
>0.70	0.77	0.43, 1.29	0.76	0.46, 1.19	0.78	0.49, 1.21
Mean rainfall during transmission season	1.27	1.03, 1.56	1.25	1.00, 1.54	1.20	0.96, 1.51
Maximum LST	0.92	0.75, 1.12	0.91	0.75, 1.10	0.89	0.72, 1.09
Distance to rivers (m)						
<500	1.00		1.00		1.00	
500-999	1.19	0.76, 1.80	1.08	0.73, 1.56	1.01	0.98, 1.55
≥ 1000	0.73	0.46, 1.10	0.71	0.46, 1.08	0.61	0.39, 0.92
*r*^c^	0.16	0.15, 0.17	0.16	0.15, 0.17	0.16	0.15, 0.17
*ρ*_1_^d^	0.0016	0.0005, 0.002	0.0013	0.0002, 0.002	0.0014	0.0003, 0.003
*ρ*_2_			0.0016	0.0005, 0.002	0.0016	0.0006, 0.003
*ρ*_3_			0.0014	0.0002, 0.002		
*σ*_1_^2e^	0.33	0.18, 0.54	0.18	0.03, 0.45	0.14	0.02, 0.37
*σ*_2_^2^			1.08	0.37, 2.73	0.60	0.27, 1.19
*σ*_3_^2^			0.26	0.06, 0.68		
**DIC^f^**	**46,046.4**		**46,044.6**		**46,045**	

^These models were used for calculation of model performance and prediction.^

^a^IRR: incidence-rate ratio; ^b^BCI: Bayesian credible interval; ^c^*r*: over-dispersion parameter; ^d^*ρ*: scalar parameter representing the rate of decline of correlation with distance between points; ^e^*σ*^2^: estimate of geographic variability; ^f^DIC: deviance information criterion; a composite measure of how well the model does, i.e. a compromise between fit and complexity, with smaller DICs indicating better performance of the model.

**Table 4:** Percentage of test individuals with *P. falciparum *parasitaemia falling within selected Bayesian credible intervals (BCIs).

BCIs	Bayesian negative binomial regression model		
	
	Stationary	Non-stationary (ecological subregions)	Non-stationary (fixed subregions)
95%	99%	99%	99%
75%	94%	93%	93%
50%	63%	63%	64%
25%	14%	13%	13%
5%	2%	1%	2%
4%	1%	1%	1%
3%	1%	1%	1%
2%	1%	1%	1%
1%	0%	0%	0%

### Risk mapping

**Figure 2:**
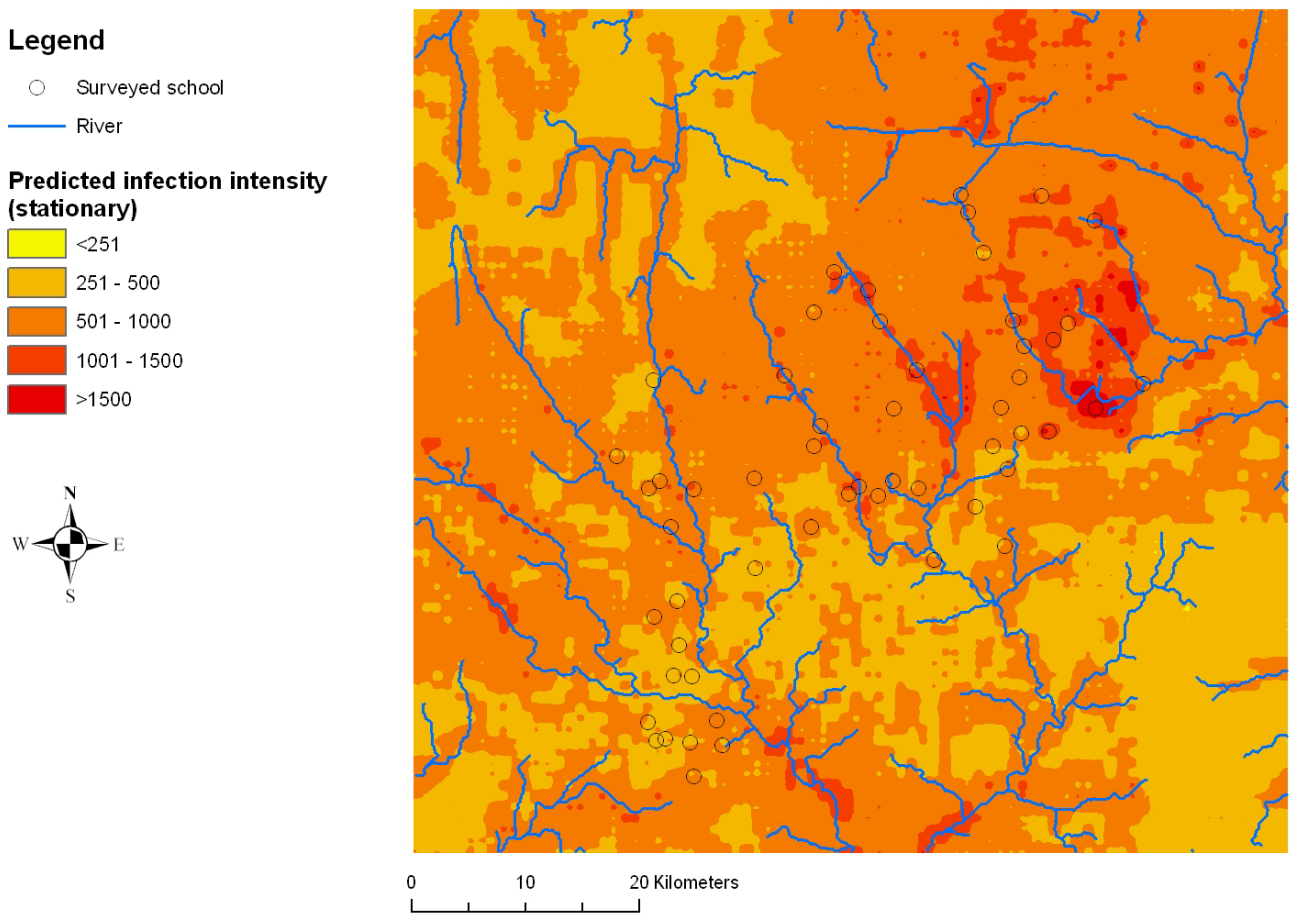
**Smoothed map of *P. falciparum *parasitaemia derived from a stationary negative binomial regression model using Bayesian in the region of Man, western Côte d'Ivoire**.

**Figure 3:**
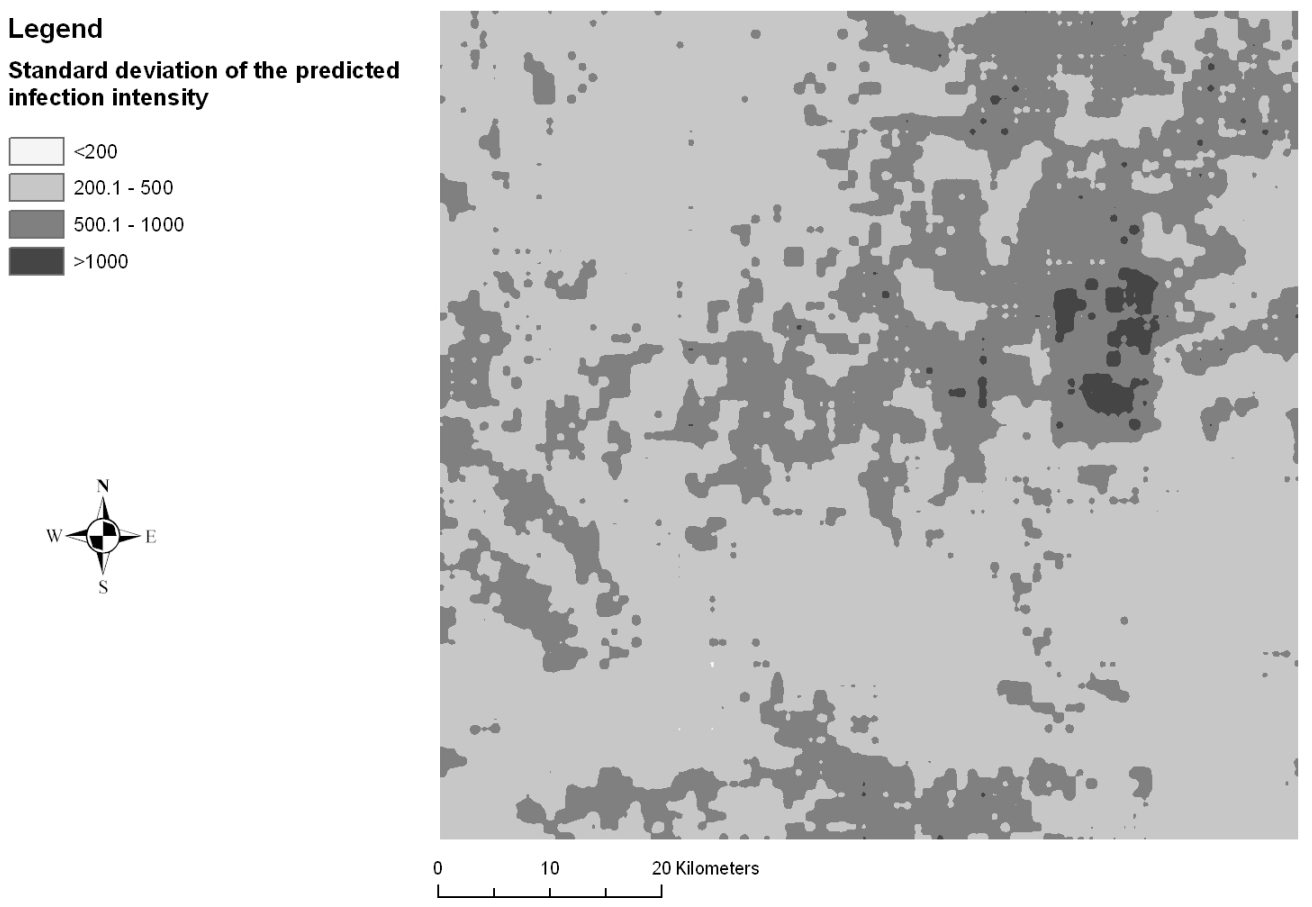
**Standard deviation map of the predicted *P. falciparum *parasitaemia derived from a stationary negative binomial regression model using Bayesian kriging in the region of Man, western Côte d'Ivoire**.

**Figure 4:**
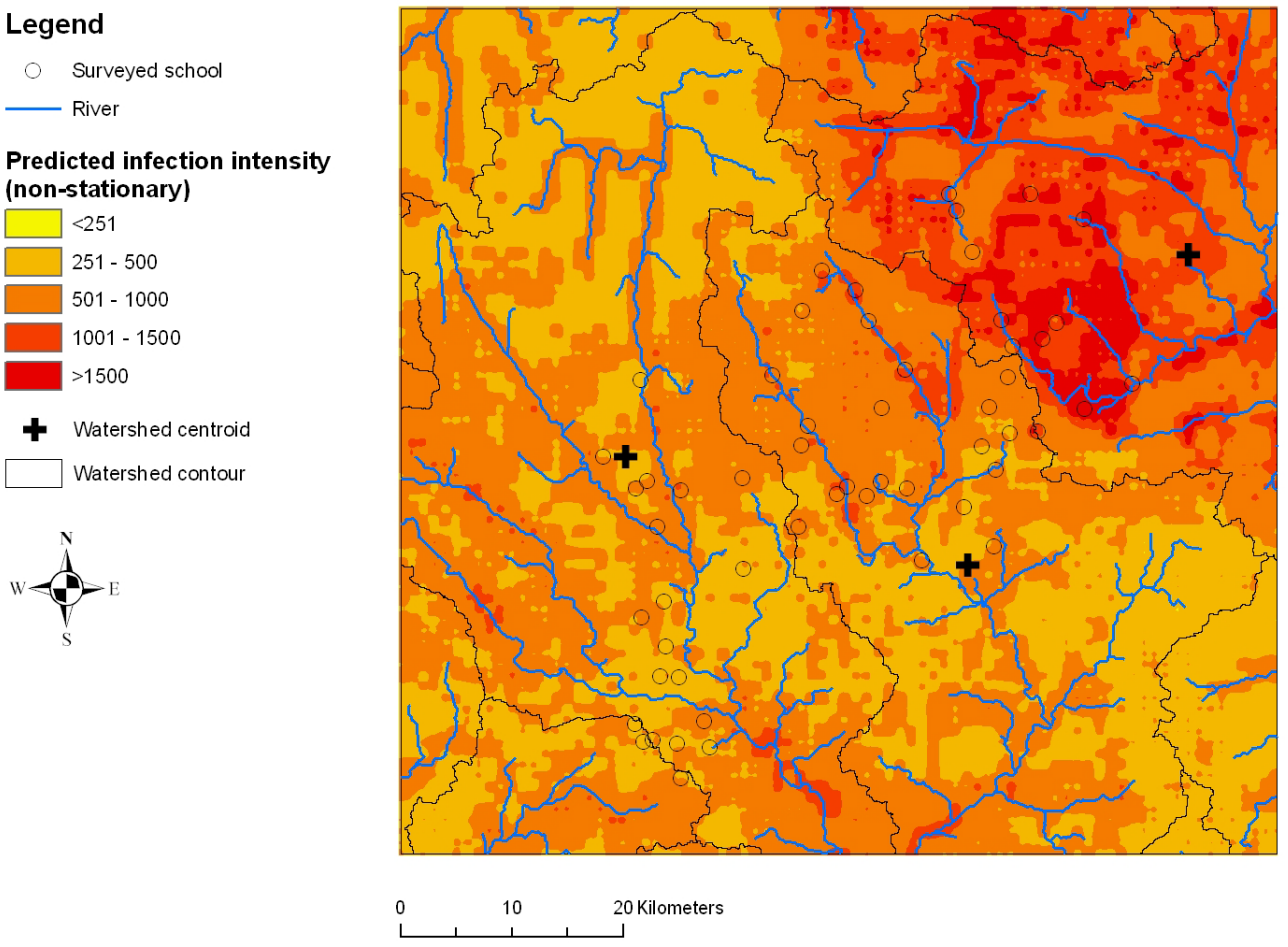
**Smoothed map of *P. falciparum *parasitaemia derived from a non-stationary negative binomial regression model with ecologic subregions using Bayesian kriging in the region of Man, western Côte d'Ivoire**.

**Figure 5:**
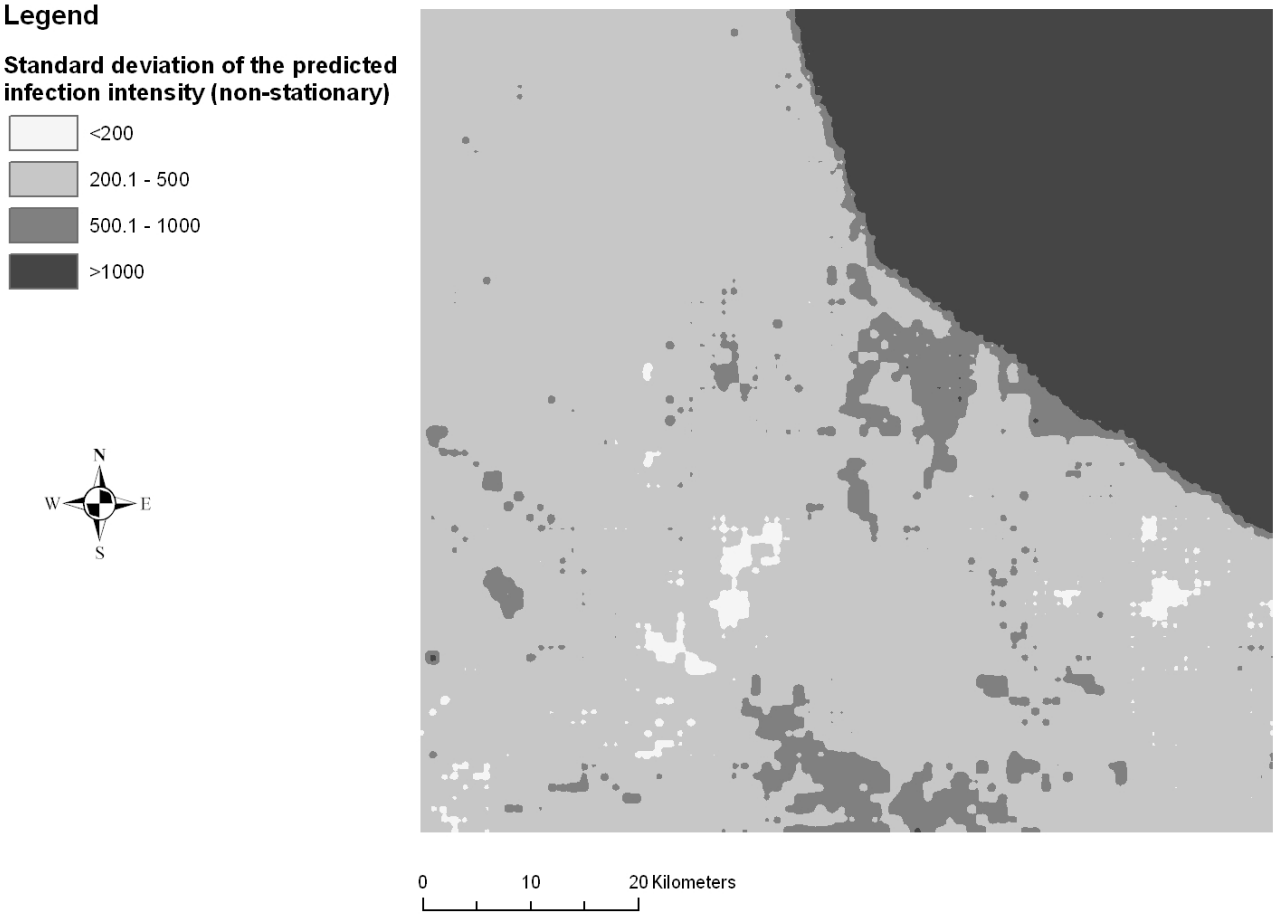
**Standard deviation map of the predicted *P. falciparum *parasitaemia derived from a non-stationary negative binomial regression model with ecologic subregions using Bayesian kriging in the region of Man, western Côte d'Ivoire**.

**Figure 6:**
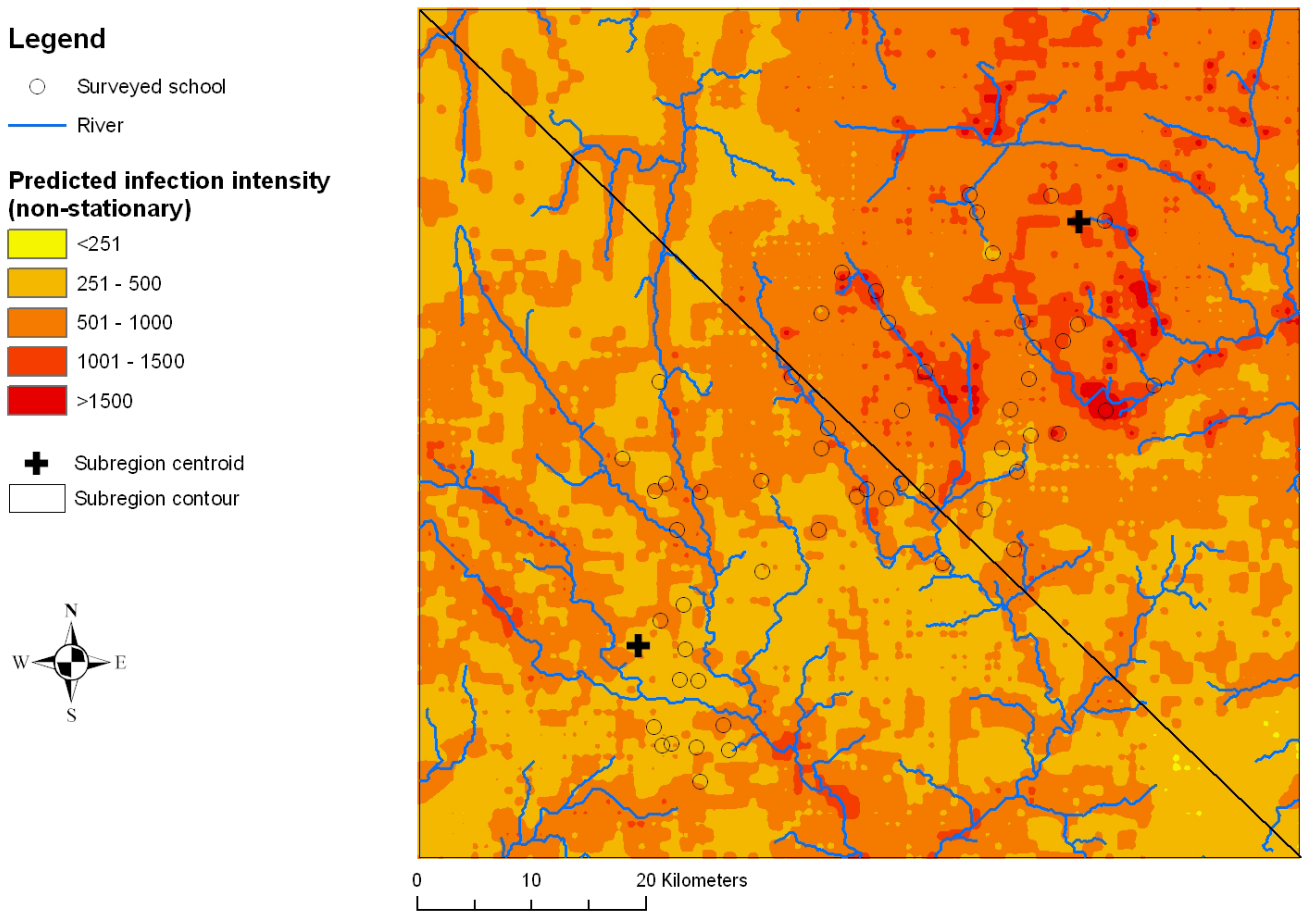
**Smoothed map of *P. falciparum *parasitaemia derived from a non-stationary negative binomial regression model with fixed subregions using Bayesian kriging in the region of Man, western Côte d'Ivoire**.

**Figure 7:**
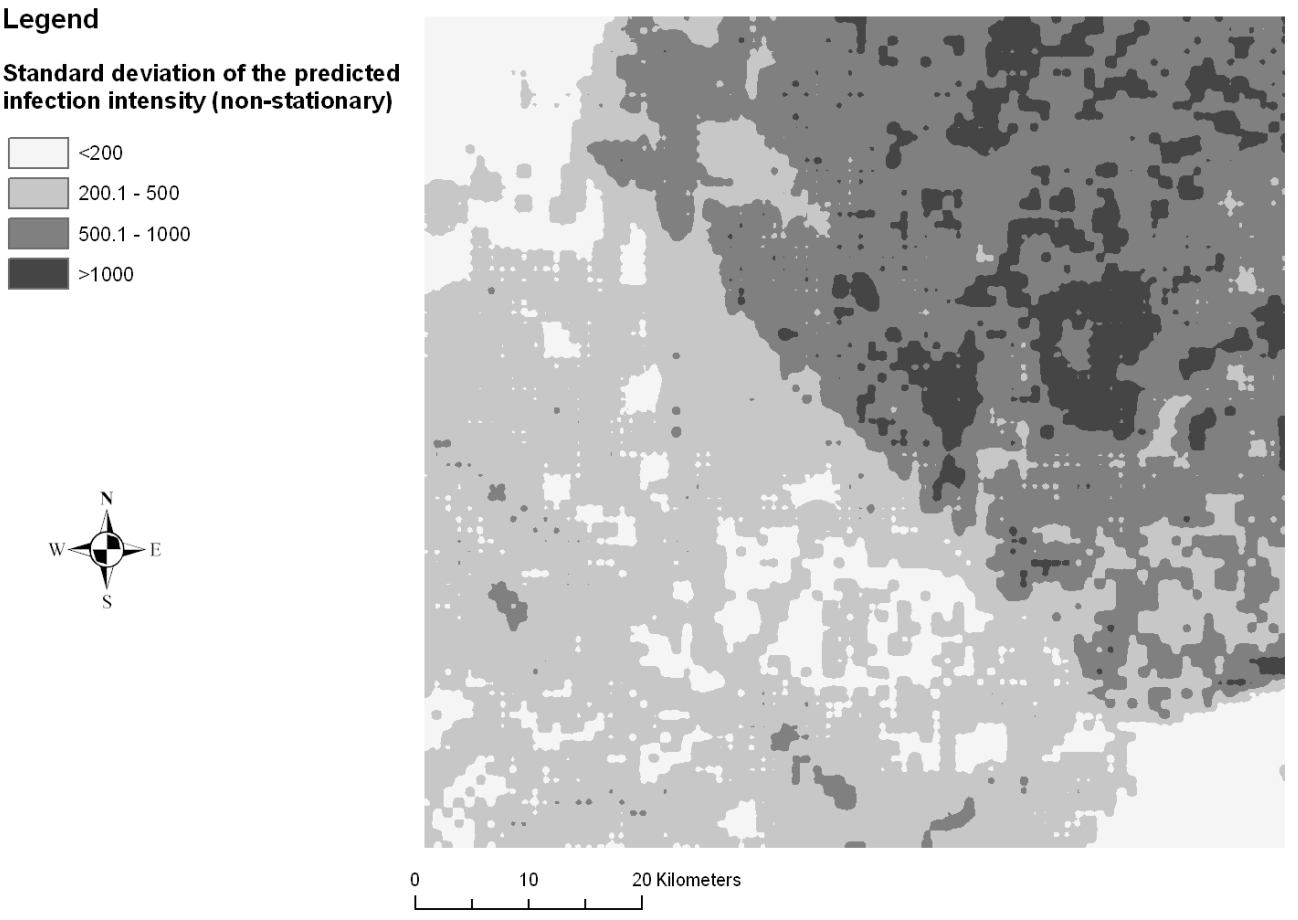
**Standard deviation of the predicted *P. falciparum *parasitaemia derived from a non-stationary negative binomial regression model with fixed subregions using Bayesian kriging in the region of Man, western Côte d'Ivoire**.

Figures 2, 3, 4, 5, 6 and 7 display the results from the three stationary and non-stationary *P. falciparum *parasitaemia models. The maps were based on models without the covariates bed net coverage, presence of swamps, distance to irrigated fields and presence of pasture, as this information was missing for prediction. There is a clear difference in the parasitaemia predictions between stationary and non-stationary models. In the non-stationary map inferred from the non-stationary model with ecological subregions, the predicted parasitaemia was considerably higher in the north-eastern part of the study area compared to the maps derived from the stationary model and the non-stationary model with fixed subregions. However, the standard deviations of the predicted parasitaemia inferred from the non-stationary model with ecological subregions show that in this area the prediction error is highest. Nonetheless, all three standard deviation maps show increased standard errors in the north-eastern part of the Man area in western Côte d'Ivoire.

## Discussion

Current anti-malarial prophylaxis and treatment, and vector control using insecticides are susceptible to the emergence of resistant malarial parasites and vectors. Hence, there is a pressing need for other interventions incorporated into the programme that can delay the onset of resistance. There is also a need for new drugs and insecticides and a malaria vaccine, coupled with improved monitoring and surveillance [28]. Mapping areas where people are at an elevated risk of infection and *P. falciparum *parasitaemia is important for the design and implementation of district-based malaria control interventions.

Here an integrated approach for spatial risk profiling of *P. falciparum *parasitaemia was used, building on previous research pertaining to the mapping and prediction of helminth infections in the Man region, western Côte d'Ivoire [29]. Reasons why this approach is termed 'integrated' are as follows. First, a diversity of data (demographic, environmental and socioeconomic) was obtained from different sources, including cross-sectional questionnaire and epidemiological surveys and remote sensing. Second, data covered different spatial scales. For example, RFE, LST and NDVI data were collected by remote sensing at a large spatial scale. At a small spatial scale, data on proximity to standing water (e.g., swamps and irrigated agricultural fields) were obtained from questionnaires addressed to school directors and from digitized maps. Third, the data were collated, stored and managed using a GIS. Finally, Bayesian geostatistical models were employed to produce smoothed risk maps of *P. falciparum *parasitaemia, and to compare model outcomes assuming either stationary or non-stationary dependence. Age, socioeconomic status, sleeping under a bed net, bed net coverage and different environmental factors - both small-scale (e.g., close proximity to standing water) and large-scale (e.g., LST, NDVI and RFE) - were significant risk factors for *P. falciparum *parasitaemia. Interestingly, after introducing spatial correlation into the regression analyses, age, bed net coverage and - depending on the type of the model - mean RFE over the malaria transmission season, and distance to rivers appeared to be significant risk factors for *P. falciparum *parasitaemia. Appraisal of model performance revealed no difference when comparing stationary with non-stationary models. However, the non-stationary model with ecological subregions showed that the geographical variability is different between subregions.

Two shortcomings of the present study should be noted. First, school-aged children are usually not the most severely affected group with malaria in highly endemic areas. Since the western part of Côte d'Ivoire is holoendemic for malaria [15,17,18,21], it is likely that school-aged children have acquired some kind of immunity to malarial parasites [30,31]. However, parasitaemia levels in school-aged children might be higher than in younger children. Underlying reasons are that school-aged children in high endemicity areas are mainly asymptomatic carriers, they might be more exposed to mosquito bites due to their behaviour, they are less likely to be treated because of a lower incidence of clinical malaria, and hence they might harbour considerably more parasites than preschool-aged children. Second, due to the possibility of sequestration mechanisms of infected erythrocytes from peripheral blood, as well as partially acquired immunity, microscopic examination of only a single finger prick blood sample might have underestimated the true prevalence of infection, and *P. falciparum *parasitaemia might have been slightly different [32-34].

Notwithstanding these shortcomings, several risk factors were found to be associated with *P. falciparum *parasitaemia, including demographic factors (e.g., age), socioeconomic factors, personal preventive measures (e.g., sleeping under a bed net and bed net coverage) and a host of environmental factors. As expected, children who reported sleeping under a bed net were less likely to have a high malaria parasitaemia as were children from schools with a bed net coverage >25%. A study from rural Tanzania revealed that people from poorer households were less likely to access preventive measures [35]. A similar result has been reported for the population under study here [19]. Based on these observations and the common belief that the poorest population segments would share the highest burden of malaria, the current results surprisingly point in the opposite direction: schoolchildren from better-off households were more likely to have a higher parasitaemia than their poorer peers. This result is in accordance with previous work focusing on spatial risk profiles of *P. falciparum *prevalence in the same group of children [15] and consequently warrants further investigation.

For the current mapping of *P. falciparum *parasitaemia, a similar geostatistical approach was used as before when modeling *P. falciparum *prevalence data [15] and common helminth infections [22,23,36]. Importantly, the statistical significance of several covariates changed once spatial correlation had been taken into account. For example, children's socioeconomic status, sleeping under a bed net and several environmental factors - most notably LST, NDVI, close proximity to standing water and presence of pasture - were not significant anymore in the spatial models. This issue might be explained because omission of spatial correlation, when analysing spatially-explicit data, overestimates the significance of the regression coefficients [13]. In contrast to previous spatial analyses of *P. falciparum *prevalence data, it was found that environmental factors such as rainfall during the main malaria transmission season and distance to the nearest permanent river were significant predictors for *P. falciparum *parasitaemia. These environmental covariates are related to the presence and abundance of malaria vectors, including *Anopheles gambiae *and *Anopheles funestus*, which are the key vector species as found in previous work in the nearby forest and wet Savannah zones of Côte d'Ivoire [37,38] and the medium-sized town of Man located in the centre of the current study area [20]. As shown in a study from Burkina Faso these vectors breed in small pools (*An. gambiae*) and larger semi-permanent water bodies (*An. funestus*) [39]. In previous research pertaining to *P. falciparum *prevalence data, most of the environmental factors included had a large spatial scale and none of the environmental covariates was found significant [15]. Hence, it was concluded that environmental data at a small spatial scale are necessary for more precise spatial risk profiling at the district level where decisions are usually made for the control of malaria and other infectious diseases. Indeed, including information obtained from interviewing the directors of schools about the proximity of residential houses to standing water revealed a number of significant environmental covariates in the non-spatial analyses, although there was a lack of statistical significance in the spatial models. At a more local or regional scale, only distance to rivers, which was used as a proxy for standing water, was significant in one of the spatially-explicit models. Further ground-based investigations are required, since only data derived from questionnaires and digitized maps were used rather than ecological surveys to explore small-scale environmental features. It will also be interesting to determine the use of topography-derived wetness indices, which have been linked to household malaria risk at small spatial scale in two communities in the Kenyan highlands [40]. Perhaps somewhat surprising at first, the present spatial analyses showed that RFE during the main malaria transmission season, which is rather a broad scale indicator, indicated the spatial heterogeneity of parasitaemia in the study area. This observation might be explained by the distinct climatic conditions, i.e., higher precipitation in the mountainous northern part of the study area.

Comparing the performance of different models did not reveal any significant difference in the predictive ability between stationary and non-stationary models, and hence the predicted parasitaemia risk maps were similar. Interestingly though, the non-stationary model with ecological subregions predicted a slightly larger area with high parasitaemia in the north-eastern part of the study area. The corresponding standard deviations of the map showed that uncertainty was particularly high in this subregion. A likely explanation of this observation is that there were fewer sampled locations in that specific subregion (Figure 1). However, uncertainty in the north-eastern part of the study area was also elevated (though to a lesser extent) when employing a stationary and a non-stationary model with fixed subregions. Of note, the spatial parameters in the non-stationary model with ecologic subregions revealed that geographic variability differed between subregions. Consequently, this would rule in favour of using non-stationary models for predicting *P. falciparum *parasitaemia. Previous spatial analyses of *P. falciparum *prevalence in the same area revealed that non-stationary models performed somewhat better than stationary models [15].

An important aspect of the current study is that the statistical model approach influences not only the spatial parameter estimates, including the prediction maps and standard deviations of the prediction, but also the significance of malaria risk indicators. Depending on the statistical model chosen, i.e., stationary or non-stationary, the significance of several environmental factors changed. For example, in the stationary and the non-stationary models with ecological subregions, mean RFE during the main malaria transmission season was significantly explaining the geographical heterogeneity, whereas in the non-stationary model with fixed subregions, this covariate was not significant. Instead, distance to rivers appeared as a significant covariate in the non-stationary model with fixed subregions. Such differing results have also been reported by others when comparing stationary and non-stationary models for the risk of malaria across Mali [9]. The covariate mean RFE during the main malaria transmission season had the lower BCIs near 1 in both stationary and non-stationary models with ecological subregions, and the increase in odds due to increased rainfall was only 0.28 and 0.24, respectively. In contrast, the non-stationary model with fixed subregions seems particularly promising, as the upper BCI for the covariate distance to rivers was not close to 1 and the parasitaemia risk decreased by over a third with increasing distance from rivers.

Employing a spatially-explicit risk profiling approach, demographic, environmental and socioeconomic risk factors were identified that govern the geographic distribution of *P. falciparum *parasitaemia in a high endemicity area at the district level. This information can be utilized for designing and implementing malaria control interventions. In particular, at the time of the study in 2001/2002, virtually no malaria control interventions were carried out in the region of Man. The very low frequency of schoolchildren reported sleeping under a bed net (< 10%) documents this issue [19]. Although bed nets were available for purchase from local dispensaries and the district hospital in the town of Man, the price was perceived as too high. It is speculated that the malaria situation in this region has not improved, partially explained by an armed conflict starting in September 2002 that also hit the region of Man and resulted in a collapse of the health care delivery systems [41,42]. Available information supports this claim; coverage of bed nets (ITNs) was reported below 5% in Côte d'Ivoire at a national scale [43] and in the Man region in particular [44]. The results further suggest that health-seeking regarding prevention and treatment of malaria at dispensaries was weak, as no statistical significance was found with regards to distance to a health post.

## Conclusion

A massive scale-up of bed net coverage in the region of Man, ideally promoting LLINs is indicated. Villages located in the north-eastern part of the study area and those in close proximity to rivers should be targeted first to have the strongest impact. Once control interventions will start to take off, it is conceivable that the malaria situation will become more heterogeneous across the Man region, and hence stationarity in modeling prevalence and parasitaemia will no longer be justified, as control interventions are likely to vary depending on location. Future field studies will elucidate whether the presented integrated risk profiling and control approach can also be employed for rigorous monitoring and performance evaluations of the district-level malaria control programme.

## List of abbreviations

ACT: Artemisinin-based Combination Therapy; ADDS: Africa Data Dissemination Service; AIC: Akaike Information Criterion; BCI: Bayesian Credible Interval; CI: Confidence Interval; DALYs: Disability-Adjusted Life Years; DEM: Digital Elevation Model; DIC: Deviance Information Criterion; GIS: Geographical Information system; GPS: Global Positioning System; ITN: Insecticide-Treated Nets; LLINs: Long-Lasting Insecticidal Nets; LST: Land Surface Temperature; MCMC: Markov Chain Monte Carlo; MODIS: Moderate Resolution Imaging Spectroradiometer; NDVI: Normalized Difference Vegetation Index; RFE: Rainfall Estimates; SRTM: Shuttle Radar Topography Mission.

## Competing interests

The authors declare that they have no competing interests.

## Authors' contributions

GR contributed to the conception and design, participated in the data collection, carried out the spatial analyses and interpretation of the data and drafted the manuscript. KDS was involved in the data collection, quality control, data analyses and drafting of the manuscript. PV contributed to the analysis of the data and drafting of the manuscript. BHS was involved in the interpretation of the data and critical revision of the manuscript. AY was involved in the acquisition of data. MT contributed to the conception and design. JU contributed to the conception and design, interpretation of the data and drafting of the manuscript. EKN was involved in the conception and design as well as the critical revision of the manuscript. All authors read and approved the initial submission and the revised version of the manuscript.
